# Targeted Expression of Cre Recombinase Provokes Placental-Specific DNA Recombination in Transgenic Mice

**DOI:** 10.1371/journal.pone.0029236

**Published:** 2012-02-17

**Authors:** Cissy Chenyi Zhou, Jiang Chang, Tiejuan Mi, Shahrzad Abbasi, Dongmin Gu, Le Huang, WenZheng Zhang, Rodney E. Kellems, Robert J. Schwartz, Yang Xia

**Affiliations:** 1 Departments of Biochemistry and Molecular Biology, Texas A&M Health Science Center, Houston, Texas, United States of America; 2 Institute of Bioscience and Technology, Texas A&M Health Science Center, Houston, Texas, United States of America; 3 Internal Medicine, University of Texas Medical School at Houston, Houston, Texas, United States of America; Brigham and Women's Hospital, United States of America

## Abstract

**Background:**

Inadequate placental development is associated with a high incidence of early embryonic lethality and serious pregnancy disorders in both humans and mice. However, the lack of well-defined trophoblast-specific gene regulatory elements has hampered investigations regarding the role of specific genes in placental development and fetal growth.

**Principal Findings:**

By random assembly of placental enhancers from two previously characterized genes, trophoblast specific protein α (*Tpbpa*) and adenosine deaminase (*Ada*), we identified a chimeric *Tpbpa/Ada* enhancer that when combined with the basal *Ada* promoter provided the highest luciferase activity in cultured human trophoblast cells, in comparison with non-trophoblast cell lines. We used this chimeric enhancer arrangement to drive the expression of a *Cre* recombinase transgene in the placentas of transgenic mice. *Cre* transgene expression occurred throughout the placenta but not in maternal organs examined or in the fetus.

**Significance:**

In conclusion, we have provided both *in vitro* and *in vivo* evidence for a novel genetic system to achieve placental transgene expression by the use of a chimeric *Tpbpa*/*Ada* enhancer driven transgene. The availability of this expression vector provides transgenic opportunities to direct the production of desired proteins to the placenta.

## Introduction

The mammalian placenta is the first organ to be developed during gestation and carries out multiple functions required for normal embryonic development in the uterine environment [1]. Impaired placental development is associated with many complications to both moms and babies during pregnancy, including preeclampsia, intrauterine growth retardation (IUGR) and fetal loss [2], [3]. Thus, a better understanding of gene function during placentation could provide new insights regarding normal placental development and fetal growth, which will in turn help guide the development of prevention strategies and new therapies for the treatment of diseases associated with pregnancy, including fetal abnormalities.

Genetic manipulation of the mouse is a powerful experimental approach to study the functional role of specific genes by gain of function or loss of function strategies [4]. However, the disruption of many genes results in embryonic lethality because of placental defects, making it difficult to evaluate the potential role the gene may play in extraplacental tissues [1]. In addition, the embryonic lethality of these mutant mice prevents their use to assess the role of specific genes in postnatal physiology and development. Over the past decade, the *Cre/loxP* system, utilizing Cre recombinase to catalyze a deletion event between two DNA fragments containing the 34 bp *loxP* recognition site, has been commonly used for conditional gene deletion strategies to assess biological function of genes in certain types of cells or tissues *in vivo* [5]. However, the lack of a robust placental specific transgene to efficiently and specifically express desired genes (including *Cre* recombinase) in placenta has hampered progress in a number of areas of placental developmental biology. For example, the inability to disrupt the expression of specific genes in the placenta has prevented the analysis of the exact role of these genes in placental development. In addition, the inability to reliably direct expression of transgenes to the placenta has prevented the use of transgenic strategies to correct placental defects, thereby making it possible to study the role of a specific gene in other (i.e., non-placental) aspects of embryonic development and/or postnatal development. To overcome this limitation, we set out to develop a mammalian expression vector containing strong placental enhancers to drive robust placental specific expression of desired genes.

To reach this goal, we focused on the placental enhancers from two genes known to drive the expression of reporter genes in placenta of transgenic mice. The first enhancer is a 340 bp regulatory fragment from the 5′-flanking region of trophoblast specific protein α gene, *Tpbpa*, a gene encoding a putative inhibitor of placental cathepsins that is specifically expressed in the trophoblast lineage in mice [6], [7], [8]. The other is a 1.8 kb fragment from the 5′ flanking region of the gene encoding adenosine deaminase (ADA), an enzyme present at high levels in trophoblast cell lineages throughout placental development [9]. Early studies identified a 1.8 kb fragment in the 5′-flanking region of the *Ada* gene that is responsible for placenta specific expression [10], [11]. However, the characterized placental regulatory elements for *Ada* generally provided relatively low placental expression. Therefore, we attempted to generate a robust placental specific enhancer by testing combinations of both *Tpbpa* and *Ada* enhancers.

## Materials and Methods

### Reagents

One-step RT-PCR kit, cell culture medium, antibiotics and fetal bovine serum (FBS) were purchased from Invitrogen (Carlsbad, CA). pCAG-CATZ and AdMA19 plasmids were a generous gift from Dr. M.D. Schneider (British Heart Foundation Centre of Research Excellence, National Heart and Lung Institute, Imperial College London, London SW7 2AZ, UK)). PKS-*Tpbpa* plasmid was a generous gift from Dr. Janet Rossant (University of Toronto, Toronto, Ontario, M5S 1A1 Canada). Cre recombination-reporter mice were purchased from Jackson laboratory. HEK-293, M1, A549, ML12, K562 and HeLa cells were purchased from the American Type Culture Collection (Manassas, VA).

### Plasmid construction

The *Tpbpa* enhancer fragment was generated by Pfu DNA polymerase-based PCR using PKS-*Tpbpa* plasmid [6] and paired primers flanked with KpnI sequence, 5′ primer, TAGGTACCGTAGACTGTTCCTCAGTAGA; 3′ primer, TAGGTACCCTCGAGAGAGAA AGACACTT. PCRs were performed with an initial denaturation at 95°C, 2 min. PCR cycling conditions were 30 cycles of denaturation at 95°C for 30 sec, annealing at 60°C for 30 sec, extension at 72°C, 60 sec, and final extension at 72°C for 10 min. The PCR product of the *Tpbpa* enhancer was digested with KpnI and subcloned into KpnI restriction site of pGL3-basic luciferase vector to generate p*Tpbpa*-Luc. In addition, the *Tpbpa* PCR fragment was blunted and subcloned into p*Ada*-Luc vector containing the 0.8 kb basal *Ada* promoter (*Ada*P) or *Ada* enhancer/*Ada*P containing the 1.8 kb *Ada* enhancer and 0.8 kb basal *Ada* promoter to generate p*Tpbpa*-*Ada*P-*Luc* and p*Tpbpa*/*Ada*-*Ada*P-Luc vector in reverse or forward direction (p*Tpbpa*r/*Ada*f-*Ada*P-*Luc* and p*Tpbpa*f/*Ada*f-*Ada*P-*Luc*). Finally, a second copy of *Tpbpa* was further subcloned into p*Tpbpa*f-*AdaP*-*Luc* or p*Tpbpa*r/*Ada*f-*Ada*P-*Luc* constructs to generate p*Tpbpa*f-*Tpbpa*f/*AdaP*-*Luc* and p*Tpbpa*f-*Tpbpa*r/*Adaf*-*Ada*P-*Luc*.

To generate *Tpbpa*r/*Ada*f-*Ada*P-Cre vector, a *Cre* DNA recombinase fragment (1.9 kb) containing a nuclear localization sequence (NLS) was released from pαMHC-Cre plasmid by digestion with *Kpn*I, subsequently blunted with T7 DNA polymerase and then cut with *Hind* III. This fragment was subcloned into p*Tpbpa*r/*Ada*f-*Ada*P-*Luc* construct with replacement of luciferase fragment by digestion with *Xba*I, blunted with T7 DNA polymerase and then cut with *Hind* III. The accuracy of all construct sequences was confirmed by DNA sequencing.

### Transient Transfection and luciferase activity

Human trophoblast cell line HTR-8/SV*neo* (HTR) [12] and non-trophoblast cell lines HEK-293 (human embryonic kidney), HeLa (human cervical carcinoma), M1 (renal tubular epithelial), A529 (human lung epithelial), ML12 (mouse lung epithelial)and K562 (human leukemia)were plated on 6-well plates at 1.0×10^5^ cells/well with RPMI1640 medium supplemented with 10% FBS and 1% antimycotic for overnight. Cells were transfected with various constructs using Fugene 6. A *Renilla* luciferase construct was used as a transfection efficiency control. After 24 hours, cellular extracts were isolated and luciferase activity measured using a dual luciferase assay kit as described (Promega, Madison, WI) [3], [13].

### PCR analysis of Cre recombinase activity in vitro

To determine Cre-mediated DNA recombination after transient transfection, PCR analysis was conducted as described [14]. Briefly, HTR cells were cotranfected with the *Cre*-dependent reporter gene, CAG-CATZ, with or without CMV-Cre or different amounts of *Tpbpa*r/*Ada*f-*Ada*P-*Cre* construct, respectively. CAG-CATZ harbors a CAT gene flanked by *loxP* sites and driven by the chicken β-actin promoter. Downstream of CAT is E coli β-galactosidase (*lacZ*). DNA was isolated 48 h after transfection and PCR analysis was performed using oligonucleotides synthesized corresponding to the 3′end of the chicken β-actin promoter (5′-CTGCTAACCATGTTCATGCC-3′; AG2) and 5′ end of the LacZ gene (5′-GGCCTCTTCGCTATTACG-3′; Z3). In addition, additional oligonucleotides synthesized corresponding to the 3′end of the CAT gene (5′-CAGTCAGTTGCTCAATGTACC-3′; CAT2) and 5′ end of the CAT gene (5′-ACTGGTGAAACTCACCCA-3′; CAT3) were used as an internal control for transfection efficiency and resulted in a 320 bp PCR fragment. Using AG2 and Z3 primers, in the absence of Cre, only the 2100 bp precursor PCR fragment was detected. However, in the presence of Cre, both 2100 bp (precursor) and 690 bp (product) PCR fragments were detected. Any cells transfected with the Cre reporter were screened by PCR using primer pair CAT2 and CAT3 which give a 320 bp PCR product.

### Luciferase analysis of Cre recombinase activity in vitro

Similar to PCR analysis of Cre recombinase activity, HTR cells were transfected with AdMA19 luciferase construct containing a spacer interposed between two *loxP* sites precluding efficient lucifearse expression in the absence of Cre recombinase. Forty-eight hr after transfection, cellular extracts were collected and luciferase activity was measured as described [3], [13], [15].

### Generation of transgenic mice

All animal manipulations in this study were reviewed and approved by the Animal Welfare Committee, University of Texas Houston Health Science Center (Protocol# HSC-AWC-09-159). The 5 kb *Tpbpa*r/*Ada*f-*Ada*P-*Cre* gene containing the *Tpbpa*r/*Ada*f-*Ada*P chimeric enhancer *Ada* promoter and Cre recombinase transgene was excised from *Tpbpa*r/*Ada*f-*Ada*P-*Cre* plasmid vector backbone using *Not*I and *Hpa*I. Linear *Tpbpa*r/*Ada*f-*Ada*P-*Cre* DNA fragment was separated by electrophoresis through 1% agrose gel and purified using Qiaex II reagents (QIAGEN Inc., Chatsworth, CA). The linear *Tpbpa*r/*Ada*f-*Ada*P-*Cre* gene was microinjected into the pronuclei of FVB zygotes [6], [16] at a concentration of 2 ng/µl in 10 mM Tris-HCl (pH 7.4), 0.1 M EDTA and injected embryos were transferred to pseudopregnant FVB females.

### RNA isolation, quantification of real time PCR (RT-qPCR) and semi-quantitative RT-PCR

Trizol reagent was used for the isolation of total RNA. The reverse transcriptase-polymerase chain reaction (RT-PCR) was performed according to the manufacturer's recommended protocol (Invitrogen, Carlsbad, CA). 1 µg of RNA was used per reaction and single strand cDNA was synthesized at 55°C for 30 minutes. The annealing temperature of PCR for Cre and GFP is 50°C. PCR conditions were as described [15]. Cre primer sequences were: sense primer, 5′-CCCTGTTTCACTATCCAGGT, and antisense primer, 5′-GGGTAACTAAACTGGTCGAG. GFP primer sequences were: reverse, 5′-GGCCATGATATAGACGTT; forward, 3′- AAGTTCATCTGCACCACCG. β-actin was used as an internal control and primer sequences were: reverse, 5′-CCACCGATCCACACAGAGTAC and forward, 5′-GCTCTGGCTCCTAGCACCAT. RT-PCR products were revealed on 2% agarose gels. RT-qPCR was performed using SYBR green JumpStart Taq ReadyMix (Sigma) on an Applied Biosystem 7000 under the following conditions: 95°C, 2 min; 95°C, 15 s; 50°C, 15 s; 72°C, 30 s; 40 cycles. Each cDNA sample was run in triplicate. Relative Cre expression was calculated following normalization to β-actin.

### Genomic DNA isolation, genotyping by PCR and gene copy number analysis

Genomic DNA was isolated using a DNeasy tissue kit (Qiagen, Valencia, CA). Presence of *Tpbpa*r/*Ada*f-*Ada*P-*Cre* transgenes in founders was assessed by amplification of genomic DNA from tail samples [6], [16], using a sense primer at the 5′ end and anti-sense primer for the Cre cDNA, respectively (5′-CGGTCTCTGAGAGCCATC-3′ and 5′- CCCTGAACATGTCCATCA-3′) and resulting in a 340 bp band. To determine the genotype of placentas from pregnant F1 offspring, genomic DNA was isolated from embryos. Cre recombinase transgene gene copy number was determined by qPCR as described [17]. qPCR was performed with 200 ng of DNA in duplicate using green JumpStart Taq ReadyMix (Sigma) on an Applied Biosystem 7000. Endogenous mouse β-actin was used as an internal control for DNA input. The quantitative standard curve was generated using a series of standard samples containing 1, 2, 4, 8, 16 copies of the Cre gene respectively, which were prepared by mixing wild type DNA from FVB mice with the *Tpbpa*r/*Ada*f-*Ada*P-Cre vector. Standard curve was drawn by plotting C_tCre_ against the log of *Cre* gene copies of corresponding standard samples. *Cre* was amplified from genomic DNA extract from transgenic mouse tissues and the gene copy number was obtained by using the standard curve with the given sample C_tCre_.

### Cre recombinase and ADA immunostaining

Mouse tissues were fixed in 10% formalin overnight, dehydrated, paraffin embedded and cut at 4 µm thickness. Slides were stained using standard methods. Briefly, slides were stained with either sheep anti-mouse ADA (1∶400 dilution) [18] or rabbit anti-Cre (1∶1000 dilution, EMD4 Bioscience, Gibbstown, NJ) at 4°C overnight. Signal was detected with either anti-sheep or anti-rabbit IgG horseradish peroxidase kit (ABC kit, Vector Laboratory, Burlingame, CA). Counterstaining is hemotoxylin.

### LacZ staining and quantificaiton

Mouse placentas were bisected. Half of each placenta was frozen with liquid nitrogen and half was mounted in freezing medium and then frozen with liquid nitrogen. Sets of 4–6 µm cryostat sections were obtained and fixed in 4% formaldehyde for 10 min. 5′-bromo-4-chloro-3-indolyl- β-D galactopyranoside (X-gal) staining was performed as described [6]. The counterstain was nuclear fast red. Quantification of the LacZ staining was performed using the Image-Pro Plus software. The density of each zone of blue staining (positive for LacZ) was measured. The average densities of each zone of placentas were averaged and the SEM is indicated.

### Statistical analysis

All data are expressed as the mean ± SEM. Statistical significance of the differences between the mean values of multiple groups was tested by one-way ANOVA, followed by Tukey-Kramer post-tests. Data were analyzed for statistical significance using GraphPad Prism 4 software (GraphPad Software, San Diego, CA). A value of *P*<0.05 was considered significant.

## Results

### Random assembly of *Tpbpa* and *Ada* placental enhancers upstream of the *Ada* basal promoter to drive expression of a luciferase reporter gene

**Figure 1:**
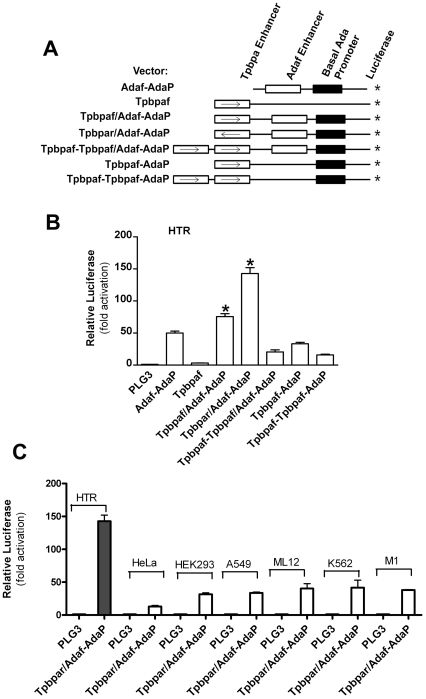
Random assembly of placental specific enhancers and *in vitro* analysis of their ability to activate the basal promoter of the *Ada* gene in multiple cell types. (**A**) Schematic illustrations of the constructs. (**B**) Luciferase activity in human trophoblast (HTR) cells transfected with each construct. (**C**) Luciferase activity driven by *Tpbpa*r/*Ada*f-*Ada*P chimeric enhancer in multiple cell lines. Data are expressed as mean ± SEM. n = 4–6. * *P*<0.05 versus cells transfected with *Ada*f-*Ada*P construct.

*Tpbpa* and *Ada* placental enhancers are among the few enhancers known to confer placenta-specific gene expression *in vivo*. However, the utility of these enhancers has been limited by low expression levels. Thus, to generate a potentially stronger placental expression vector we chose to randomly assemble placental enhancers from the *Tpbpa* and *Ada* genes in front of the *Ada* basal promoter (for details see Methods and Materials section). To quantify the expression of these chimeric enhancer promoter combinations, a luciferease reporter vector was used as shown in Figure 1A.

### Screening for expression constructs with trophoblast specific transcriptional activity

The various constructs were analyzed by transfection into the human trophoblast cell line (HTR) followed by the measurement of luciferase activity in cell extracts. All of the constructs exhibited enhanced luciferase expression in the trophoblast cell line except the enhancerless basal luciferase vector and a construct lacking the *Ada* basal promoter (Figure 1B). Among all of the constructs tested, a particular construct in which the *Ada* enhancer was in the forward orientation and the *Tpbpa* enhancer was in the reverse orientation (*Tpbpa*r/*Ada*f-*Ada*P) showed the highest luciferase activity in the trophoblast cell line (Figure 1B). We also tested this construct for luciferase expression in a variety of non-trophoblast cells, including HEK293 (renal cell), Hela (cervical carcinoma), M1 (renal tubular epithelial cell), A529 (human lung epithelial cell), ML12 (mouse lung epithelial cell) and K562 (human leukemia cell). Notably, the luciferase activity driven by the double enhancer construct, *Tpbpa*r/*Ada*f-*Ada*P, was significantly higher in trophoblast cells (HTR) than in non-trophoblast cells (Figure 1C), suggesting that this construct is capable of enhanced expression in trophoblast cells. Thus, the *Tpbpa*r/*Ada*f enhancer combination provoked the highest transcriptional activity among the constructs tested and provided gene expression that was restricted to trophoblast cells.

### *Tpbpa/Ada* enhancer construct drives Cre-mediated recombination in cultured trophoblast cells

**Figure 2:**
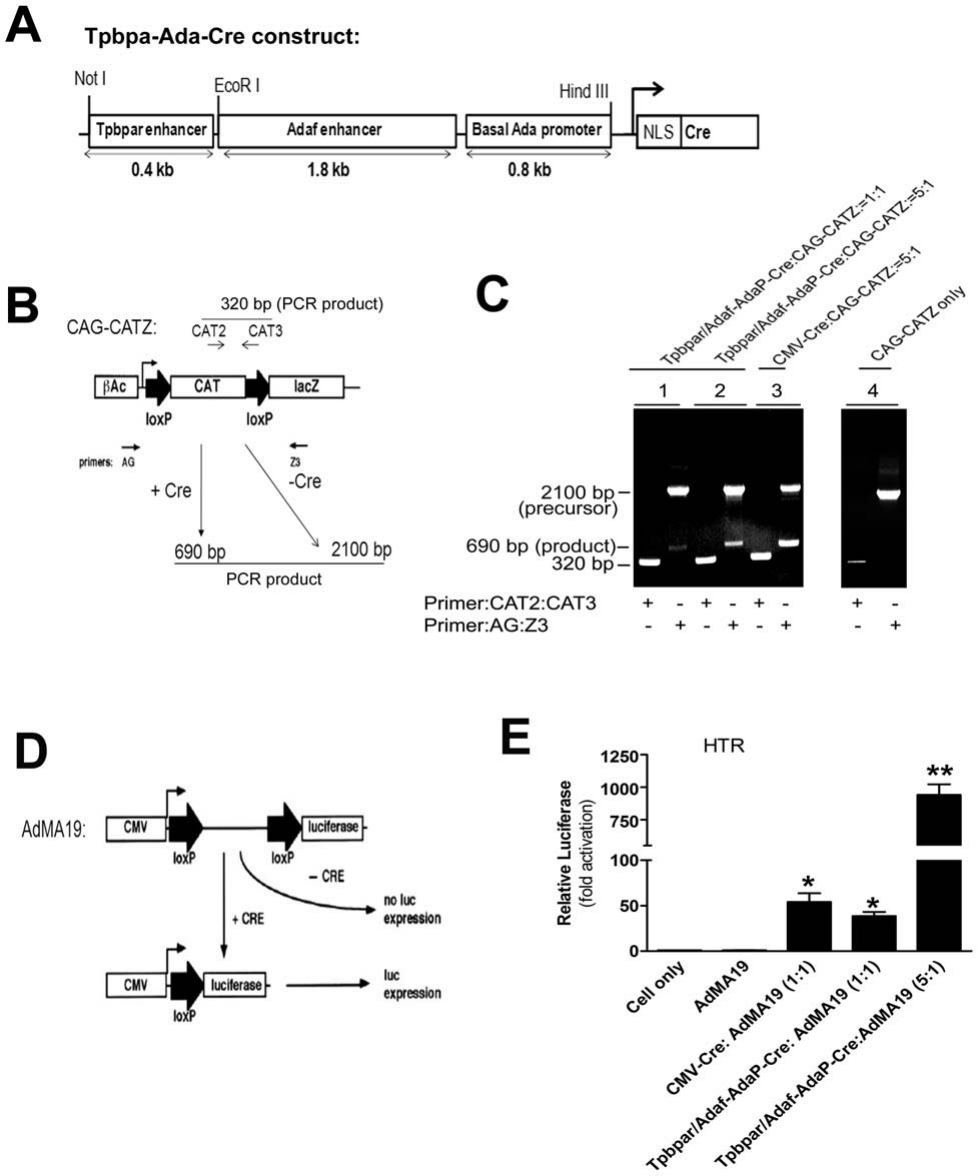
Generation of *Tpbpa*r/*Ada*f-*Ada*P-*Cre* chimeric expression vector and *in vitro* analysis of its Cre recombinase activity in human trophoblast cells. (**A**) Structure of p*Tpbpa*r/*Ada*f-*Ada*P-*Cre* construct. *Tpbpa*r/*Ada*f chimeric enhancer and *Ada* basal promoter (*Ada*P) were ligated to the sequence encoding Cre cDNA containing a nuclear localization signal (NLS). (**B**) Schematic representation of pCAG-CATZ vector. The PCR primers, primer pair 1 (AG and Z3) were used to monitor Cre-mediated loxP-dependent DNA recombination (2100 bp for parental DNA, 690 bp for the recombined DNA). Primer pair 2 (CAT2 and CAT3) were internal primers used to detect pCAG-CATZ. (**C**) PCR analysis: pCAG-CATZ was transfected alone or together with CMV-Cre or different amounts of *Tpbpa*r/*Ada*f-*Ada*P-*Cre* into human trophoblast cells (HTR). DNA was isolated 48 h after transfection and assayed for the presence of the recombination-dependent 690 bp fragment. In the absence of Cre, only the 2100 bp precursor PCR fragment was observed. However, in the presence of Cre, both the 2100 bp precursorand the 690 bp product PCR fragments were detected. The amount of 690 bp PCR fragment observed increased with additional *Tpbpa*r/*Ada*f-*Ada*P-*Cre* transfected to the cells. The 320 bp PCR fragment was used to determine that pCAG-CATZ was transfected into the cells. (**D**) Schematic representation of AdMA19 vector. Spacer interposed between the *loxP* sites precludes efficient lucifearse expression in the absence of the Cre recombinase. (**E**) Luciferase analysis. AdMA19 vector was transfected with CMV-*Cre (CMV-Cre/AdMA19*,1∶1), different amounts of *Tpbpa*r/*Ada*f-*Ada*P-*Cre* (*Tpbpa*r/*Ada*f-*Ada*P-*Cre/AdMA19,1∶1 or 5∶1*) or alone. Cellular extracts were isolated 48 h after transfection and luciferase activity was measured. All data are expressed as mean ± SEM. n = 6. * *P*<0.05 versus cells transfected with AdMA19 construct only. **P<0.05 versus Tpbpar/*Ada*f-*Ada*P-*Cre/AdMA19,1∶1*.

The *Cre/loxP* system provides a powerful investigative strategy to study gene function in specific types of cells or tissues. To achieve trophoblast-restricted expression of Cre recombinase, we modified the *Tpbpa*r/*Ada*f-*Ada*P expression construct by replacement of the luciferase reporter gene with cDNA encoding *Cre* recombinase equipped with a nuclear localization signal (NLS) (Figure 2A). Next, the ability of the *Tpbpa*r/*Ada*f-*Ada*P-*Cre* construct to direct the synthesis of *Cre* recombinase in trophoblast cells was determined by a *lox*P site specific recombination assay as described previously [14]. HTR cells were co-tranfected with *Cre*-dependent reporter genes, CAG-CATZ for PCR analysis or AdMA19 for luciferase bioassay, together with CMV-Cre or different amounts of *Tpbpa*r/*Ada*f-*Ada*P-Cre transgene (Figure 2 B & D). The CAG-CATZ plasmid harbors a CAT gene flanked by *loxP* sites and driven by the chicken β-actin promoter. Downstream of CAT is the E coli β-galactosidase gene (*lacZ*) (Figure 2B). The AdMA19 reporter gene contains a CMV promoter driving a luciferase transgene. However, efficient expression of the luciferase reporter depends on a *Cre*-mediated *lox*P recombinational event that removes of a spacer region separating the CMV promoter from the luciferase coding region (Figure 2D). A 320-bp band was detected by PCR (using internal control primers, CAT2 and CAT3) from all cells transfected with CAG-CATZ. A 690 bp PCR product (representing recombination between the two loxP sites) was detected by primers AG and Z3 when using DNA isolated from cells cotransfected with either CMV-*Cre* or *Tpbpa*r/*Ada*f-*Ada*P-*Cre* construct and *loxP* construct (CAG-CATZ). Only the 2100 bp PCR product, representing the original unrecombined DNA sequence, was detected when cells were transfected with CAG-CATZ alone (Figure 2C).

Similarly, luciferase activity was only observed in the cells transfected with either CMV-*Cre* or *Tpbpa*r/*Ada*f-*Ada*P-*Cre* and AdMA19 but not AdMA19 alone (Figure 2D). Thus, both assays indicated that Cre recombinase functions in a dosage-dependent manner to promote *loxP*-dependent DNA recombination. Notably, the lucifearse activity mediated by the Cre recombinase in human trophoblast cells transfected with the *Tpbpa*r/*Ada*f-*Ada*P double enhancer construct was even higher than that achieved by transfection with the CMV promoter driven Cre. Thus, these findings demonstrate the *Tpbpa*r/*Ada*f-*Ada*P expression vector drives enhanced expression of Cre recombinase in human trophoblast cells.

### *Tpbpa*r/*Ada*f-*Ada*P-Cre transgene directs placental-restricted expression in mice

**Figure 3:**
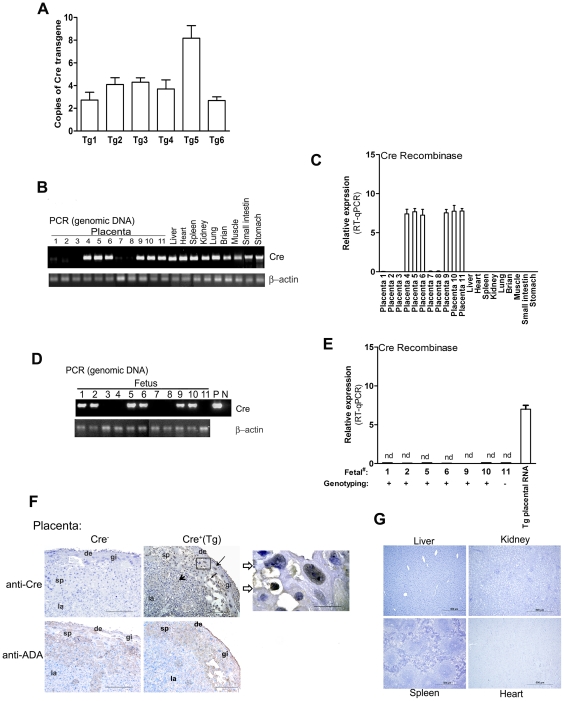
Placenta-restricted gene expression in *Tpbpa*r/*Ada*f-*Ada*P-*Cre* transgenic mice. Pregnant mice were sacrificed on gestation day 16.5 and placentas, multiple organs and embryos were collected. (**A**) Copy number of transgenes was determined by qPCR analysis in *Tpbpa*r/*Ada*f-*Ada*P-*Cre* transgenic founders. Genotyping analysis (**B**, **D**) of *Tpbpa*r/*Ada*f-*Ada*P-*Cre* transgenes by PCR and expression pattern of *Cre* mRNA (**C**, **E**) analyzed by RT-qPCR in placenta, fetus and multiple maternal organs from female transgenic mice (derived from Tg 5 founder) mated with wild type FVB male mice. β-actin was used as an internal control. Tg placental RNA is used as positive control. nd, not detectable; P, Positive control; N, Negative control. (**F**) Immunochemistry staining of Cre recombinase using anti-Cre antibody in the placentas of pregnant *Tpbpa*r*/Ada*f*-Ada*P*-Cre* females mated with wild type FVB males. Placentas with *Tpbpa*r*/Ada*f-*Ada*P-*Cre* transgenes (Cr^+^(Tg)) expressed Cre protein in giant cells (indicated by long arrow), spongiotrophoblast cells (indicated by short arrow) and cells in the labyrinthine zone (indiated by arrow head)of placentas, with highest expression in the spongiotrophoblast zone. Panel **F** (inset) showed nuclear localization of Cre in trophoblast cells. Placentas lacking *Tpbpa*r/*Ada*f-*Ada*P-Cre transgenes (Cre^−^, panel **F**) and multiple organs from pregnant transgenic dams (**G**) showed no Cre immunostaining. Endogenous ADA immunostaining was performed in placentas with or without *Tpbpa*r/*Ada*f-*Ada*P-Cre transgenes using anti-ADA antibody (panel **F**). Scale bar, 100 µm (placenta) or 50 µm (inset) and 500 µm for maternal organs.

To determine whether the chimeric *Tpbpa*r/*Ada*f-*Ada*P construct drives placenta-specific Cre recombinase expression, the construct was used to generate transgenic mice. Live births were genotyped for the *Cre* transgene. Seventeen *Tpbpa*r/*Ada*f-*Ada*P-*Cre* transgenic mice were identified from 40 pups by PCR using primers specific for the chimeric *Tpbpa*r/*Ada*f-*Ada*P construct. Eight transgenic lines were further characterized. Six out of eight transgenic lines (75%) showed the placental-restricted expression. As shown in Figure 3A, Tg 1 and Tg6 showed relatively lower copies of *Cre* transgenes than those of Tg 5 as judged by analysis of genomic DNA from tails using real time PCR. Real time PCR (qPCR) data indicated that Tg5 contains 8 copies of *Tpbpa*r/*Ada*f-*Ada*P transgene.

Next, to determine whether *Tpbpa*r/*Ada*f-*Ada*P transgenes were only expressed in the placenta we mated females from all six *Tpbpa*r/*Ada*f-*Ada*P-*Cre* transgenic lines with wild type FVB males. On gestation day 16.5 the *Tpbpa*r/*Ada*f-*Ada*P-*Cre* transgenic females were sacrificed and placentas, fetuses and multiple maternal organs were collected. We found that *Cre* mRNA was expressed in approximately half of the placentas and was not detected in any of the maternal organs tested or in fetuses of *Tpbpa*r/*Adaf*-*Ada*P-*Cre* transgenic pregnant mice. All six transgenic lines showed very similar transgene expression patterns. The expression pattern observed for one transgenic female (line 5, with the highest copy-number of the transgenes) is presented in Figure 3C and 3D. Notably, we confirmed that all placentas positive for *Cre* mRNA also contained the *Tpbpa*r/*Ada*f-*Ada*P-*Cre* transgenes, while the placentas negative for *Cre* mRNA were also negative for the transgene (Figure 3B &C). Although the genotyping analysis by PCR showed fetuses contained the *Tpbpa*r/*Ada*f-*Ada*P-*Cre* transgene, no *Cre* mRNA was detected in those fetuses (Figure 3D & E). Thus, *Tpbpa*r/*Ada*f-*Ada*P-*Cre* constructs appear to confer placental specific *Cre* mRNA expression.

To validate our RT-qPCR results, we used immunochemical analysis to determine the expression profile of Cre recombinase in the placenta and multiple other organs obtained from pregnant *Tpbpa*r/*Ada*f-*Ada*P-*Cre* transgenic mice. Immunochemistry staining by anti-Cre antibody confirmed that Cre recombinase was not observed in the liver, kidneys, heart or spleens of pregnant female transgenic mice (Figure 3G). However, Cre recombinase was observed in placentas containing *Tpbpa*r*/Adaf-Ada*P*-Cre* transgenes (Cr^+^(Tg)) but not in those lacking this trangene (Cre^−^) (Figure 3F). ADA (adenosine deaminase), a well-known trophoblast marker, is used to identify trophoblast cells in placenta. Consistent with previous studies, ADA was expressed throughout the placenta but with significantly higher expression in spongiotrophoblast region (Figure 3F). In placentas showing *Cre* mRNA expression, we found that Cre protein was present in giant cells (indicated by long arrow), spongiotrophoblast cells (indicated by short arrow) and cells in labyrinthine zone (indicated by arrow head), with highest expression in the spongiotrophoblast zone (Figure 3F). Of particular note, Cre recombinase was observed inside of the nuclei of trophoblast cells (Figure 3F, inset). Overall, our studies provide *in vivo* evidence that *Tpbpa*r/*Ada*f-*Ada*P chimeric constructs drive the expression of Cre recombinase in placentas of transgenic mice.

### Characterization of *Tpbpa*r/*Ada*f-*Ada*P-*Cre* transgene through pregnancy

**Figure 4:**
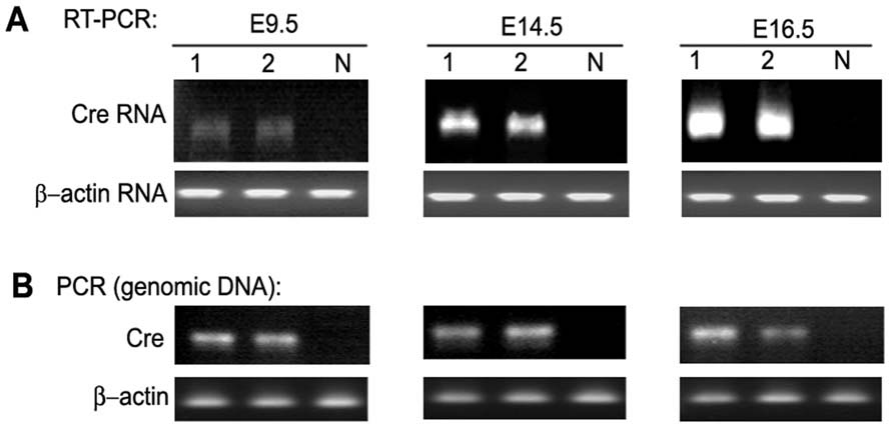
Characterization of *Tpbpa*r/*Ada*f-*Ada*P-*Cre* transgene expression at different times during pregnancy. **Pregnant mice were sacrificed on days E9.5, E14.5 and E16.5 and placentas were collected.** (**A**) RT-PCR was used to analyze the expression levels of *Cre* and β-actin mRNA in the placentas. Representative expression patterns of *Cre* and β-actin mRNA in placentas (lane 1 and 2) are shown. N, negative control placenta without transgenic Cre mRNA. (**B**) The presence of the *Cre* transgene was assessed by PCR analysis of *Tpbpa*r/*Ada*f-*Ada*P-*Cre* DNA in placentas of pregnant mice. β-actin DNA was used as an internal control.

Next, we determined *Tpbpa*r/*Ada*f-*Ada*P-*Cre* transgene expression at multiple time points of pregnancy., We observed *Cre* transgene expression in placentas as early as E9.5, with expression increasing through E14.5 and E16.5 (Figure 4 A–B). Thus, transgene expression appears to display a pattern of increasing expression from E9.5 through E16.5 and presumably reflects an increase in trophoblast cell number.

### Assessment of Cre recombinase activity in *Tpbpa*r/*Ada*f-*Ada*P-*Cre* transgenic mice

**Figure 5:**
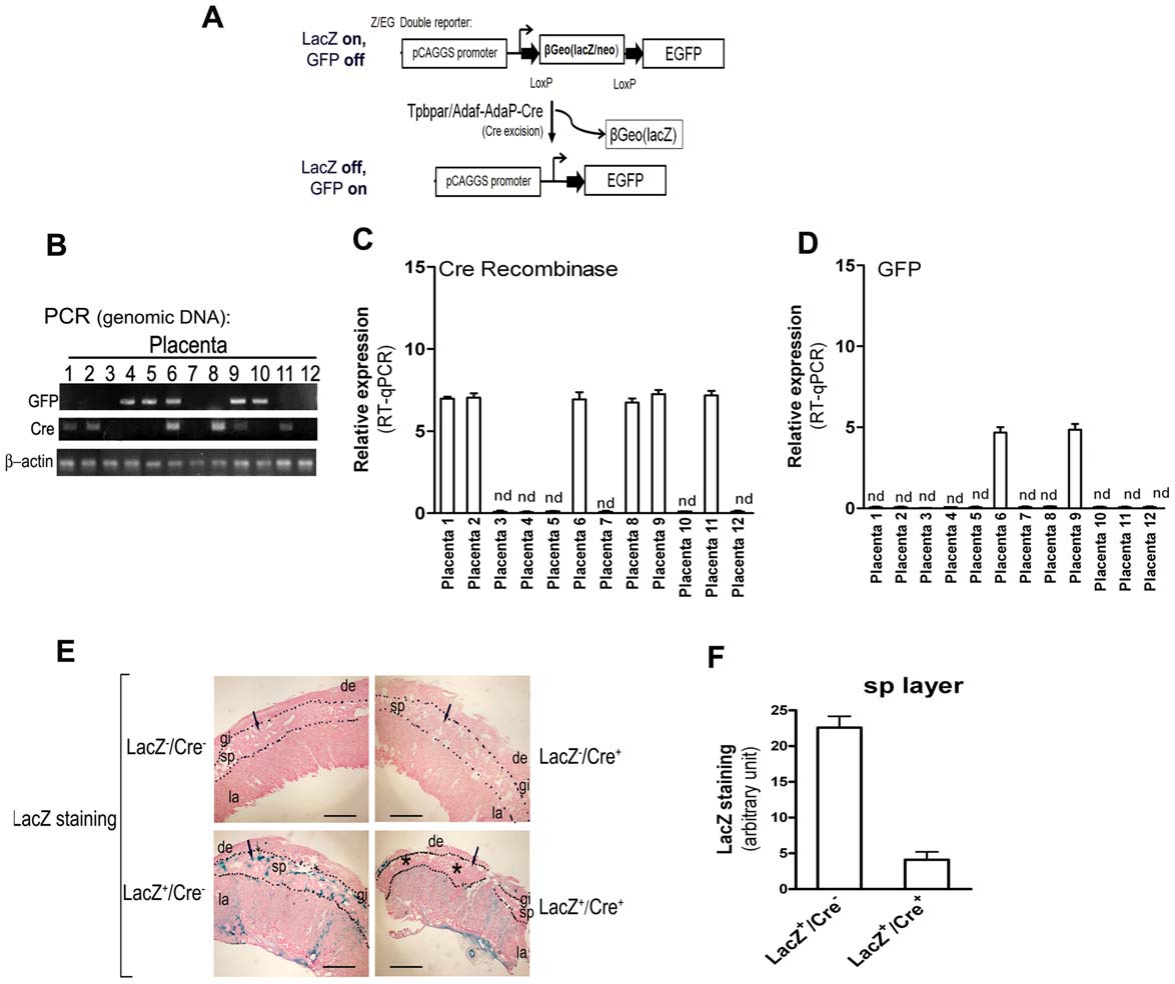
Placental-restricted DNA recombination in female *Tpbpa*r/*Ada*f-*Ada*P-*Cre* transgenic mice mated with male Z/EG double-reporter transgenic mice. (**A**) Schematic representation of *Tpbpa*r/*Ada*f-*Ada*P-*Cre* female mating with Z/EG transgenic male, double reporter mice. (**B**) *Tpbpa*r/*Ada*f-*Ada*P-*Cre* female transgenic mice were mated with Z/EG transgenic mice. On gestation E16.5, pregnant *Tpbpa*r/*Ada*f-*Ada*P-*Cre* mice were sacrificed and embryos and placentas were isolated. To define the genotype of each placenta, embryonic DNA was analyzed for the presence of *Tpbpa*r*/Ada*f-*Ada*P-*Cre* and *Z/EG* by PCR. β-actin was used as an internal control. (**C**, **D**) Expression patterns of *Cre* mRNA and GFP mRNA analyzed by RT-qPCR. nd, not detectable. (**E**) X-gal staining of multiple placentas from *Tpbpa*r/*Ada*f-*Ada*P-*Cre* pregnant transgenic mice. Nuclear fast red was used for counterstaining. Large arrows indicate the junctional zone and small arrows indicate giant cells. Scale bar: 1 mm. (**F**) Quantification of LacZ staining in spongiotrophoblast cell layer (sp layer). de, decidual cells; sp, spongiotrophoblast cells; gi, giant cells, la, labyrinth zone.

To evaluate the Cre-dependent DNA recombination in *Tpbpa*r/*Ada*f-*Ada*P-*Cre* transgenic mice, female transgenic mice were mated with Z/EG male mice, a double reporter mouse line that expresses enhanced GFP upon Cre-mediated excision of *lacZ* (Figure 5A). Pregnant mice were sacrificed at E16.5, placentas and multiple maternal organs were collected for gene expression analysis and histological studies. The results showed (Figure 5C) *Cre* mRNA was detected in approximately half of the placentas where it was correlated with the presence of the *Tpbpa*r/*Ada*f-*Ada*P-*Cre* genotype (Figure 5B). However, *Cre* mRNA in other maternal organs was not detected (data not shown). These results provide additional evidence that *Tpbpa*r/*Ada*f-*Ada* transgenes induce placental specific *Cre* expression in transgenic mice. Because Cre-mediated excision of the *lacZ* gene allows expression of the *GFP* reporter, *GFP* mRNA was only detected in the placentas that carried both *Tpbpar/Adaf-AdaP-Cre* and *lacZ-GFP* transgenes (Figure 5B, D lanes 6 and 9), while the placentas carrying only the *lacZ-GFP* transgenes or the *Tpbpar/Adaf-AdaP-Cre* trangene (LacZ^−^/Cre^+^) were negative for *GFP* mRNA (Figure 5B, D). This finding demonstrates that the *in vivo* placenta-specific expression of Cre recombinase results in *lox*P-dependent DNA recombination.

Next, histochemical staining for lacZ was used to determine Cre-mediated loxP-dependent DNA recombination at the cellular level. Consistent with our RT-PCR analysis, lacZ staining showed that constitutive expression of the lacZ reporter (blue staining) was significantly decreased in the spongiotroblast zone of placentas with lacZ^+^/Cre^+^ genotype (Figure 5E) compared with placentas having only the *lacZ^+^* transgene alone. Because of high expression of Cre in spongiotrophoblast cells layer seen in *Tpbpa*r/*Ada*f-*Ada*P-*Cre* transgenic mice (Figure 3F), lacZ quantification showed the greatest decrease in these cells (Figure 5E). Quantification of lacZ staining in spongiotrophoblast cell layer was showed as Figure 5F. Taken together, these data provide *in vivo* evidence that *Tpbpa*r/*Ada*f-*Ada*P-*Cre* transgenic mice with placenta specific expression of Cre recombinase are capable of conducting *loxP* mediated DNA recombination *in vivo*.

## Discussion

Here we report the development and characterization of a *Tpbpa*/*Ada*-*Ada*P chimeric enhancer transgene that confers a high level of trophoblast specific expression in cultured cells and in transgenic mice. Using this double enhancer construct to drive the expression of Cre recombinase, we demonstrated Cre-mediated *loxP*-dependent DNA recombination in the placenta but not in the maternal organs tested or in fetuses of transgenic mice. This double enhancer construct should be a useful genetic tool to manipulate placental gene expression in mice. This placenta expression vector should provide investigative opportunities to understand the functional role of specific genes in placental development and placenta-related pregnancy disorders.

Numerous earlier reports have attempted to identify and characterize placental gene regulatory elements. Several groups [19], [20], [21], [22] have used transgenic mouse approaches to show that a 5.4∼6.0-kb promoter and 5′-flanking sequence of *HLA-G* contains trophoblast-restricted regulatory elements. However, the level of reporter gene expression in transgenic placentas at day 12.5 was relatively low and 450 times less than endogenous β-actin. Analysis of the murine *Ada* gene by Shi *et al* [10] showed that a placenta regulatory element resided within a 1.8 kb segment of DNA in the 5′ flanking region and that this element provided consistent but variable placenta-specific expression. A trophoblast specific enhancer derived from the 5′ flanking region of the *Tpbpa* gene was also inconsistent in driving placenta specific expression in transgenic mice. Calzonetti *et al* [6] showed that a 340 bp fragment provided placenta specific expression of a lacZ reporter gene in only 5 of 16 transgenic lines (31.2%) examined. In recent years, gene transfer strategies, aimed at targeting genes to the trophoblast lineages, have been used in efforts to overcome this limitation. For example, direct injection of gene-therapy vectors into placentas results in limited levels of gene expression in the placenta, but also results in serious injury and patchy expression [23], [24]. Trophoblast-specific gene manipulation using lentivirus-based vectors has recently been developed and used in mice and rats [25], [26]. However, the lentivirus-based vector mediated trophoblast-specific gene expression requires blastocyst isolation, incubation with lentivirus vectors and the microinjection of transduced blastocysts into pseudopregnant mice [25], [26]. This approach is expensive, time consuming and inconvenient for general laboratory use. Thus, development of efficient, noninvasive and convenient genetic tools to specifically manipulate gene expression in placenta is desperately needed and would greatly facilitate efforts to understand placental formation and fetal development.

In order to construct a more robust and reliable placenta specific expression construct, we assembled a chimeric placental expression vector using placental enhancer elements from two genes, *Tpbpa* and *Ada*. Each of these genes has been previously characterized by prenatal expression in the placenta, with highest expression occurring in the spongiotrophoblast layer. Using this combinatorial strategy, we identified a novel enhancer combination containing *Tpbpa* and *Ada* regulatory elements driving transcription from the *Ada*-basal promoter. We prepared transgenes using a combination of *Tpbpa* and *Ada* enhancer elements. From seventeen transgenic mice were identified eight transgenic lines were developed by mating with nontransgenic FVB mice. Six of eight transgenic lines (75%) revealed Cre expression in placenta specifically. Thus this chimeric *Tpbpa*r/*Ada*f-*Ada*P construct showed more robust and reliable placenta specific transcription activity in transgenic mice.

We assembled a chimeric placental expression vector using placental enhancer elements from the *Tpbpa* and *Ada* genes. We have used the newly characterized expression construct to achieve placenta specific *loxP*-dependent DNA recombination mediated by a *Tpbpa/Ada* chimeric enhancer transgene encoding Cre recombinase. Notably, this chimeric enhancer construct is capable of driving *cre* gene expression in the placenta as early as E9.5, and continued expression through 16.5. Cre recombinase was observed in giant cells, spongiotrophoblasts and labyrinthine region in *Tpbpa*r/*Ada*f-*Ada*P-*Cre* transgenic mice. However, highest expression was observed in the spongiotrophoblast layer, in agreement with earlier studies of the *Tpbpa* and *Ada* placental regulatory elements.

Inadequate placenta development is associated with a high incidence of early embryonic lethality [1] and serious pregnancy disorders, such as preeclampsia [3], [27], fetal defects, fetal loss and IUGR [2], [13], [28]. Thus, for some mutant mice it is difficult to determine whether a prenatal lethal phenotype is caused by placental defects, fetal defects, or both. In addition, many gene disruptions result in embryonic lethality because of abnormal placental development and thereby prevent research opportunities to study the role of that gene in other organs prenatally and postnatally. Thus, the use of placenta specific expression constructs to drive the expression of a gene of interest in the placenta allows us to differentiate the causative factors of knockout phenotypes. For example, genetically restoring ADA enzymatic activity to placentas of *Ada*-deficient fetuses corrected most of the prenatal purine metabolic disturbances, prevented serious fetal liver damage, and rescued ADA-deficient fetuses from perinatal lethality [16]. Therefore, the placental-specific expression vector reported here is likely to provide novel possibilities to genetically restore placental expression of a gene of interest and thereby rescue embryonic lethality of mutant mice caused by placental defects.
